# Pharmacokinetic Herb-Drug Interactions of *Xiang-Sha-Liu-Jun-Zi-Tang* and Paclitaxel in Male Sprague Dawley Rats and Its Influence on Enzyme Kinetics in Human Liver Microsomes

**DOI:** 10.3389/fphar.2022.858007

**Published:** 2022-04-05

**Authors:** Alinafe Magret Kapelemera, Yow-Shieng Uang, Li-Hsuan Wang, Tien-Yuan Wu, Fang-Yu Lee, Li Tai, Ching-Chiung Wang, Chia-Jung Lee

**Affiliations:** ^1^ PhD Program in Clinical Drug Development of Herbal Medicine, Taipei Medical University, Taipei, Taiwan; ^2^ Graduate Institute of Pharmacognosy, Taipei Medical University, Taipei, Taiwan; ^3^ Rosetta Pharmamate Co., Ltd, New Taipei City, Taiwan; ^4^ School of Pharmacy, Taipei Medical University, Taipei, Taiwan; ^5^ Department of Pharmacology, School of Medicine, College of Medicine, Tzu Chi University, Hualien, Taiwan; ^6^ Traditional Herbal Medicine Research Center, Taipei Medical University Hospital, Taipei, Taiwan

**Keywords:** traditional Chinese medicine formula, Xiang-Sha-Liu-Jun-Zi tang, paclitaxel, pharmacokinetics, enzyme kinetics

## Abstract

Paclitaxel is a prescribed anticancer drug used to treat various cancers. It is a substrate of cytochrome P-450 (CYP-450) enzymes. Despite its efficacy, paclitaxel has severe side effects. Herbal medicines are commonly used to treat the side effects of chemotherapy. They can be administered before, during, and after chemotherapy. Xiang-Sha-Liu-Jun-Zi Tang (XSLJZT) is a herbal formula commonly used in breast cancer patients. The main purpose of this study was to assess the pharmacokinetic (PK) influence of XSLJZT on paclitaxel PK parameters, determine its effect on CYP-450 enzyme expression, and evaluate its effect on enzyme activity. Sprague Dawley rats were classified into pretreatment and co-treatment groups, where XSLJZT was pre-administered for 3, 5, and 7 days and co-administered 2 h before paclitaxel administration. The rat liver tissues and Hep-G2 cells were used to determine the effects of XSLJZT on CYP3A1/2 and CYP3A4 enzymes respectively. Western blot analysis was used to detect changes in the CYP3A1/2 and CYP3A4 enzymes expression. The influence of XSLJZT on enzyme activity was evaluated using human liver microsomes, and a liquid chromatography-tandem mass spectrometric system was developed to monitor paclitaxel levels in rat plasma. Results demonstrated that XSLJZT increased the area under the concentration versus time curve (AUC) for paclitaxel in pretreatment groups by 2-, 3-, and 4-fold after 3, 5, and 7 days, respectively. In contrast, no significant change in the AUC was observed in the co-treatment group. However, the half-life was prolonged in all groups from 17.11 min to a maximum of 37.56 min. XSLJZT inhibited CYP3A1/2 expression in the rat liver tissues and CYP3A4 enzymes in Hep-G2 cells in a time-dependent manner, with the highest inhibition observed after 7 days of pretreatment in rat liver tissues. In the enzyme kinetics study, XSLJZT inhibited enzyme activity in a competitive dose-dependent manner. In conclusion, there is a potential interaction between XSLJZT and paclitaxel at different co-treatment and pretreatment time points.

## 1 Introduction

Paclitaxel is an antineoplastic drug used for the first-line treatment of both early-stage and metastatic cancers. It has excellent anticancer activity against a wide range of solid tumors, including breast, ovarian, lung, and colorectal cancers (Gallego-Jara et al., 2020). It inhibits the depolarization of microtubules and arrests cell division during the metaphase. Despite its efficacy, paclitaxel has a low bioavailability. It is classified as a class IV drug according to the biopharmaceutical classification, with poor aqueous solubility (<1 μg/ml) and low oral bioavailability (<2%). To improve bioavailability, paclitaxel is administered intravenously, which, unfortunately, is associated with several side effects including gastrointestinal (GI) toxicities such as nausea, vomiting, diarrhea, and stomatitis (Boussios et al., 2012; Tekade et al., 2013; Bernabeu et al., 2017).

In order to reduce the side effects of chemotherapy, most cancer patients opt to use herbal medicines. In Taiwan, over 80% of cancer patients use herbal medicines as adjuvants for chemotherapy (Lo et al., 2012; Cheng et al., 2018), to improve their quality of life and general health, prevent recurrence, and reduce the side effects of chemotherapy (Koçaşlı and Demircan, 2017). Herbal medicines can be used before, during, and after chemotherapy. Koçaşlı et al. reported that 38.9% of cancer patients used herbal medicines before surgery and chemotherapy to fight cancer and 54.1% used herbal medicines during chemotherapy to improve their quality of life and reduce the side effects. Some cancer patients use herbal medicines before and during chemotherapy for reasons that are not related to chemotherapy (Smith et al., 2014; Koçaşlı and Demircan, 2017; Lee et al., 2021).

Xiang-Sha-Liu-Jun-Zi Tang (XSLJZT) is a herbal formula commonly prescribed with chemotherapy. It is the second most commonly prescribed herbal formula after Jia-Wei-Xiao-Yao-San (JWXYS) in breast cancer patients (Lai et al., 2012; Lee et al., 2014). It consists of eight herbal constituents and originates from the herbal formula Liu-Jun-Zi Tang (LJZT) with the addition of *Amomum villosum* and *Aucklandia lappa*. The addition of these two herbs to the LJZT herbal formula promotes Qi circulation and improves GI motility and gastric emptying (Xiao et al., 2012; Shih et al., 2019). Clinically, XSLJZT is used for the treatment of spleen deficiency and Qi stagnation syndrome according to the Chinese Society of Digestive Diseases. Thus, it is one of the herbal medicines commonly prescribed in cancer patients to reduce chemotherapy-induced GI disturbances, including nausea, vomiting, abdominal distension, and diarrhea (Huang et al., 2017; Xiao et al., 2021).

Although herbal medicines are beneficial for cancer patients, their use should be closely monitored (Ohnishi and Takeda, 2015). The use of herbal medicines before and during chemotherapy can result in toxic, allergic, and carcinogenic effects due to herb-drug interactions (Koçaşlı and Demircan, 2017). Herb-drug interactions occur when some phytochemicals inhibit or induce the drug-metabolizing enzymes, which can in turn affect the dose-effect relationship of the co-administered drugs (Fasinu and Rapp, 2019). Cytochrome P-450 (CYP-450) enzymes include different subfamilies depending on the gene sequences (McDonnell and Dang, 2013; Zanger and Schwab, 2013). Paclitaxel is a substrate of CYP-450 enzymes, specifically CYP3A4 and CYP2C8 subfamilies in humans. Some studies have reported that the major metabolite of paclitaxel in the human liver is 6α-hydroxypaclitaxel (6α-OHP), which is formed by CYP2C8 and is seconded by C3′-hydroxypaclitaxel (C3′α-OHP) formed by CYP3A4. In contrast to humans, C3′α-OHP is the major metabolite in the rat liver, while 6α-OHP, the major metabolite in the human liver, is not formed in the rat liver (Václavíková et al., 2003; Vaclavikova, et al., 2004). The inhibition or induction of CYP2C8 or CYP3A4 can affect the metabolism of paclitaxel, which may in turn affect the dose-effect relationship (Hendrikx et al., 2013). XSLJZT contains phytochemicals that can potentially induce or inhibit CYP-450 enzymes (Liu et al., 2018).

The potential influence of XSLJZT on co-administered chemotherapeutics has not been extensively studied. In this study, a liquid chromatographic tandem mass spectroscopic (LC/MS/MS) detection system was used to assess the presence of paclitaxel in biological samples. Using Sprague Dawley (SD) rats, we assessed the potential herb-drug pharmacokinetic interactions of XSLJZT with paclitaxel. XSLJZT was pretreated for several days before paclitaxel administration and co-treated with paclitaxel on the same day. The effect of XSLJZT on CYP-450 enzymes was evaluated using western blot, where CYP 3A1/2 enzymes were detected in the rat liver tissues and CYP3A4 enzymes in Hep-G2 cells. Since different species metabolize paclitaxel differently, human liver microsomes were used to assess the effects of XSLZJT on the metabolism of paclitaxel in humans (Vaclavikova, et al., 2004).

## 2 Materials and Methods

### 2.1 Chemicals and Reagents

DMSO, MTT, trypan blue, and other chemicals were purchased from Sigma. Paclitaxel (Phyxol^®^), purity >99%, was obtained from Sinphar Pharmaceutical (Yilan County, Taiwan). Dulbecco’s modified minimal essential medium (DMEM), streptomycin, penicillin, and fetal bovine serum (FBS) were obtained from Gibco BRL. Trifluoroacetic acid, methanol, and acetonitrile were used as LC/MS/MS grade reagents (Merck, Darmstadt, Germany). Anhydrous ethyl ether (AEE) was used for sample preparation (Merck, Darmstadt, Germany).

### 2.2 XSLJZT Sample Preparation

The XSLJZT prescription was based on the unified formula announced by the Committee on Chinese Medicine and Pharmacy of the Department of Health (Taipei, Taiwan). It consists of Ginseng radix (*Panax ginseng* C. A. Mey. (2.5 g), Ren She), Atractylodis macrocephalae rhizoma (*Atractylodes macrocephala* Koidz. (5 g), Bai zhu); Hoelen (*Wolfiporia cocos* (Schw.) Wolf (5 g); Fu ling), and Glycyrrhizae Radix et Rhizoma. (*Glycyrrhiza uralensis* Fisch. ex DC. (2 g) Gan cao), Citri Reticulatae Pericarpium (*Citrus reticulata* Blanco, (2 g), Chen pi), Pinelliae Rhizoma (*Pinellia ternata* (Thunb.) Makino, (2.5 g), Ban xia), Amomi Fructus (*Amomum villosum* Lour. (2 g), Sha ren), and Aucklandiae Radix (*Aucklandia costus* Falc. (2 g), Mu xiang) (Liu et al., 2017). All materials were purchased from Sun Ten Pharmaceutical (New Taipei City, Taiwan) and authenticated by a non-profit organization, Brion Research Institute of Taiwan (New Taipei City, Taiwan). Voucher specimens (No. GR-20180001 for Ginseng radix, No. AMR-20180001 for Atractylodis macrocephalae rhizoma, No. H-20180001 for Hoelen, No. GLR-20180001 for Glycyrrhizae Radix et Rhizoma, No. CR-20180001 for Citri reticulatae pericarpium, No. PR-20180001 for Pinellia rhizoma, No. AF-20180001 for Amomi Fructus and No. AR-20180001 for Aucklandiae Radix was deposited at the College of Pharmacy, Taipei Medical University. XSLJZT was prepared using the following method: total drug weight of 112 g was placed in an extractor, distilled water (10-fold) was added and boiled for 30 min until half the volume was left. This procedure was repeated once. The two extracts were combined and filtered through gauze layers. The residue was discarded, and the filtrate was lyophilized at −20°C to yield a drug powder, which was stored at 4°C. The yield of XSLJZT was 25.6%.

### 2.3 High-Performance Liquid Chromatography Analysis of Marker Substances in XSLJZT

The XSLJZT sample (0.5 g) was extracted using 20 ml of 70% methanol through ultrasonic oscillation at 25°C for 15 min and then vortexed at 160 rpm at 40°C for 40 min. The sample was then filtered through a 0.45 μm syringe filter, and a 20 μl sample was directly injected into the Waters HPLC system (Milford, MA, United States), which comprised a Waters 600 pump system, Waters 2996 Photodiode array detector, Waters 717 plus autosampler, and Sugai U-620 column oven (Wakayama City, Japan). A Cosmosil 5C18-MS-II reversed-phase column (5 μm, 4.6 mm × 250 mm, Nacalai Tesque, Japan) equipped with a Lichrospher RP-18 end-capped guard column (5 μm, 4.0 mm × 10 mm, Merck, Germany) was used as the stationary phase. The gradient elution was performed using the eluents A and B (A: acetonitrile; B: 0.1 % H_3_PO_4_) according to the following profile: 0–25 min, 19%–20% A and 81%–80% B; 25–60 min, 20%–40% A and 80%–60% B; 60–90 min, 40%–55% A and 60%–45% B; 90–100 min, 55%–60% A and 45%–40% B; 100–125 min, 19% A and 81% B. The flow rate was 1 ml/min, and the column temperature was maintained at 35°C. The following marker substances in XSLJZT were chosen according to the regulations of the Taiwan Herbal Pharmacopoeia and Pharmacopoeia of the People’s Republic of China: glycyrrhizin and liquiritin for *Glycyrrhiza uralensis*, costunolide and dehydrocostus lactone for *Aucklandia lappa*; and hesperidin for *Citrus reticulata*. We used an ultraviolet detection wavelength of 225 nm for costunolide and dehydrocostus lactone, 250 nm for glycyrrhizin, and 280 nm for liquiritin and hesperidin.

### 2.4 LC/MS/MS Conditions and Reagent Preparation for Paclitaxel Analysis

An LC/MS/MS system was used for analytical separation. It consisted of a Quattro Ultima mass spectrometer (Micromass, Manchester, United Kingdom), a Waters Alliance 2795 pump and autosampler (Waters, MA, United States), Biosil ODS 4.6 × 250 mm, 5-µm column (Biotic Chemical, Taipei, Taiwan). Data acquisition was performed using MassLynx vers. 4.0, (Micromass). The mobile phase consisted of 70% CH_3_CN and 0.1% formic acid. The flow rate was 1.00 ml/min with a post column split of 1/10 to tandem MS. The ionization mode was electrospray/positive ionization, and mass scanning was conducted in the multiple reaction monitor (MRM) mode. The paclitaxel precursor ion was detected at *m/z* 854.36, and the product ion at *m/z* 286.04. The capillary voltage was 3.2 kV, cone voltage was 35 eV, source temperature was 80°C, desolation temperature was 400°C, and collision voltage was 25 eV. A primary standard stock solution of paclitaxel (1,000 μg/ml) was prepared by dissolving 1 mg of pure paclitaxel in 1 ml methanol to prepare a standard stock solution of 1 mg/ml. A working solution was prepared by diluting the stock solution in 50% (v/v) methanol to obtain the following concentrations: three standard solutions of 10, 100, and 1,000 ng/ml, representing low-, intermediate-, and high-strength stock solutions prepared using serial dilution.

### 2.5 Method Validation

All method validation experiments were performed according to the Food and Drug Administration (FDA) guidelines (Health, U.D.o. and H. Services, 2001). The matrix effect, recovery rate, accuracy, and precision were evaluated. The liquid–liquid extraction method was used for the preparation of all biological samples.

#### 2.5.1 Matrix Effect and Recovery Rate

To determine the matrix effect and recovery rate, samples were classified into sets 1, 2, and 3. Three different concentrations (10, 100, and 1,000 ng/ml) of paclitaxel were used in each set to represent high, middle, and low concentrations. In set 1, paclitaxel was prepared in the mobile phase, while in set 2, three lots of blank plasmas were extracted and spiked with paclitaxel after the extraction. Set 3 consisted of three lots of blank plasma spiked with paclitaxel before extraction*.* Anhydrous ethyl ether was used for extraction, and all samples were evaporated to dryness after extraction, which was later reconstituted with 100 µl methanol for LC-MS/MS detection. The matrix effect was established by comparing the paclitaxel peak areas of set 2 samples to peak areas of equivalent concentrations in set 1, and the recovery rate was established by comparing the peak areas of set 3 to the corresponding peak areas in set 2.

#### 2.5.2 Calibration Curve

A calibration curve was prepared by spiking 90 µl of rat plasma with different concentrations (5–1,000 ng/ml) of paclitaxel (10 µl). Linearity was achieved at a regression coefficient of *r* ^2^ > 0.995. The limit of detection (LOD) and lower limit of quantification (LLOQ) were defined as the signal-to-noise (S/N) ratios of 3 and 10, respectively.

#### 2.5.3 Evaluation of Accuracy and Precision Using Inter-day and Intra-day Assay

The accuracy and precision were determined by quantitating three replicates of plasma samples spiked with paclitaxel at concentrations of 10, 100, and 1,000 ng/ml. The samples were prepared on the same day and on three consecutive days to represent the intra-day and inter-day samples, respectively. The accuracy was estimated using the equation: bias (%) = [(observed concentration—nominal concentration)/nominal concentration] × 100. The precision was calculated using the formula: relative standard deviation (RSD %) = [standard deviation/observed concentration] × 100.

### 2.6 Herb-Drug Pharmacokinetic Interactions

Male SD rats, provided by the Laboratory Animal Center of Taipei Medical University, were used to determine the effects of XSLJZT on the pharmacokinetics of paclitaxel. All animal experiments were done in accordance with regulations of the animal experimentation committee of Taipei Medical University (IACUC, approval no. LAC-2015-0105). The animals had a 12 h light/dark cycle and had free access to food and water. Each group consisted of six rats, and paclitaxel (2 mg/kg, i. v.) was administered to the control group, while other animals were divided into four groups according to the different treatments they received. The first treatment group (which represented co-treatment) received XSLJZT (250 mg/kg, p. o.) 2 h prior to paclitaxel (2 mg/kg, i. v.) administration, while in the second, third, and fourth groups (pretreatment), XSLJZT (250 mg/kg, p. o.) was administered for 3, 5, and 7 days prior to paclitaxel administration, respectively. Blood samples (220 μl) were collected from the jugular vein at the following time intervals: 1, 5, 10, 15, 30, 60, 120, 180, 240, 300, and 360 min following paclitaxel administration, and centrifuged at 1800 rpm for 10 min to collect the plasma. Samples were prepared using a liquid-liquid extraction method, and LC/MS/MS was used to detect paclitaxel.

### 2.7 Western Blot Analysis for Metabolic Enzymes Expression

#### 2.7.1 Preparation of Animals for Rat Liver Tissue

Male SD rats were used for liver tissue protein collection and were randomly divided into seven groups. The first group served as control group and was administered distilled water. The second, third and fourth groups were treated with XSLJZT (250 mg/kg, p. o.) only for the different treatment days (3,5, and 7 days, respectively), whereas the sixth, seventh and eighth groups received XSLJZT (250 mg/kg, p. o.) for three different pre-treatment days (3, 5, and 7 days, respectively), in addition to paclitaxel (2 mg/kg, i. v.). Liver tissues were collected, and RIPA lysis buffer was used to extract the protein from the liver tissues. The samples were then centrifuged, and the supernatants were collected for protein quantification.

#### 2.7.2 Hep-G2 Cells Preparation

Hep-G2 cells were cultured in Dulbecco’s modified Eagle’s medium (DMEM) supplemented with 10% fetal bovine serum (FBS), 1% penicillin-streptomycin, and 1% L-glutamine at 37°C in a 5% CO_2_ atmosphere. The cells were seeded in 6-well plates at a concentration of 1 × 10^5^/ml. Medium (0.2 ml) was added to each well, and the cells were incubated for 24 h. Then, the medium was discarded, and the cells were treated with different concentrations of XSLJZT for 24 h. Time dependency was evaluated by treating the cells with 800 μg/ml XSLJZT for 1, 2, 4, 6 and 24 h. After cell collection using trypsin, the protein was extracted using RIPA lysis buffer and stored at −20°C for western blot analysis (Cui et al., 2014).

#### 2.7.3 Western Blot Analysis

To determine the influence of XSLJZT on CYP3A4 enzymes in Hep-G2 cells and CYP3A1/2 in rat liver tissues, a bicinchoninic acid (BCA) assay kit was used to quantify the protein concentration. Protein samples (50 µg) were used for western blot analysis. Sodium dodecyl sulfate-polyacrylamide gels (10%) were used for protein separation and transferred onto polyvinylidene difluoride (PVDF) membranes. Non-fat milk (5%) was used to block the samples at room temperature for 1 h before adding the primary antibodies (GAPDH and CYP3A4 antibodies). This was followed by incubating the membranes overnight at 4°C. The next day, the membranes were washed with Tris buffer at room temperature for 30 min. Mouse and goat anti-rabbit IgG were used as secondary antibodies at room temperature for 1 h before visualizing the protein bands with ECL prime detection reagent, using the Chemi Doc MP imaging system (Li et al., 2021).

### 2.8 Enzyme Kinetics Using Human Liver Microsomes

To determine the influence of XSLJZT on the metabolism of paclitaxel, human liver microsomes were used (HLMs). The HLMs (0.5 mg/ml) were added with phosphate buffered saline (0.1 M, pH = 7.4) (PBS) and XSLJZT (0.5, 5, and 10 mg/ml) and paclitaxel (1, 4, 8, and 16 µM) and). The reaction was initiated by the addition of an NADPH generating system (1 mM NADP, 10 mM glucose 6-phosphate, 2 IU/ml glucose 6-phosphate dehydrogenase, and 5 mM MgCl2), and the samples were incubated for 30 min at 37°C in a shaking water bath. The reaction was terminated by the addition of acetonitrile (200 µL) and centrifugation at 18,000 × *g* for 10 min. The supernatant was extracted into new Eppendorf tubes and evaporated to dryness. The residues were then reconstituted for the analysis. Time dependency was evaluated by pre-incubating the HLMs with XSLJZT (0.5, 5, and 10 mg) for different pre-incubation times (0, 10, and 30 min). After each pre-incubation period, paclitaxel (16 µM) was added, and the samples were incubated for 30 min (Wattanachai et al., 2011; Cheng et al., 2017).

### 2.9 Data Analysis

WinNonlin standard edition version 1.1 software was used to analyze the PK parameters. Each dataset was analyzed using a two-compartment model. The PK parameters observed included the drug concentration at zero time (C_0_), the area under the concentration versus time curve (AUC), half-life (T_1/2_), clearance (CL), and mean residence time (MRT). For the statistical analysis, two groups were compared with an unpaired Student’s t-test using Sigma Plot 10.0 software. Statistical significance was set to *p* < 0.05. Data were summarized as the mean ± standard deviation.

## 3 Results

### 3.1 HPLC Analysis of Marker Substances in XSLJZT

**FIGURE 1:**
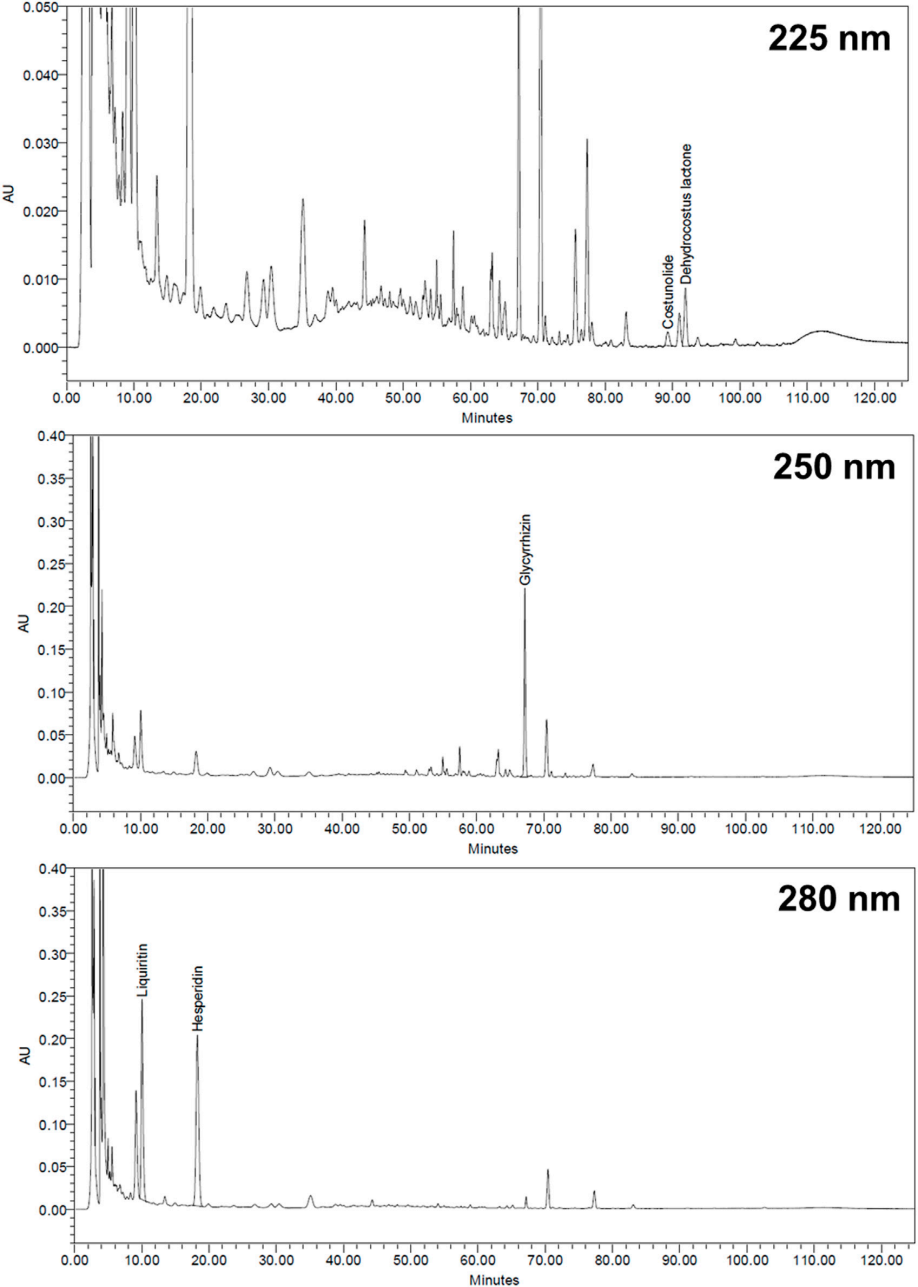
HPLC chromatogram of Xiang-Sha-Liu-Jun-Zi Tang (XSLJZT).

The retention times for liquiritin, hesperidin, glycyrrhizin, costunolide, and dehydrocostus lactone were 10.04, 18.27, 67.17, 89.25, and 91.92 min, respectively (Figure 1). Each Gram of XSLJZT contained liquiritin, hesperidin, glycyrrhizin, costunolide, and dehydrocostus lactone at 3.13, 16.86, 3.84, 0.04, and 0.15 mg, respectively.

### 3.2 LC/MS/MS Method Validation

**FIGURE 2:**
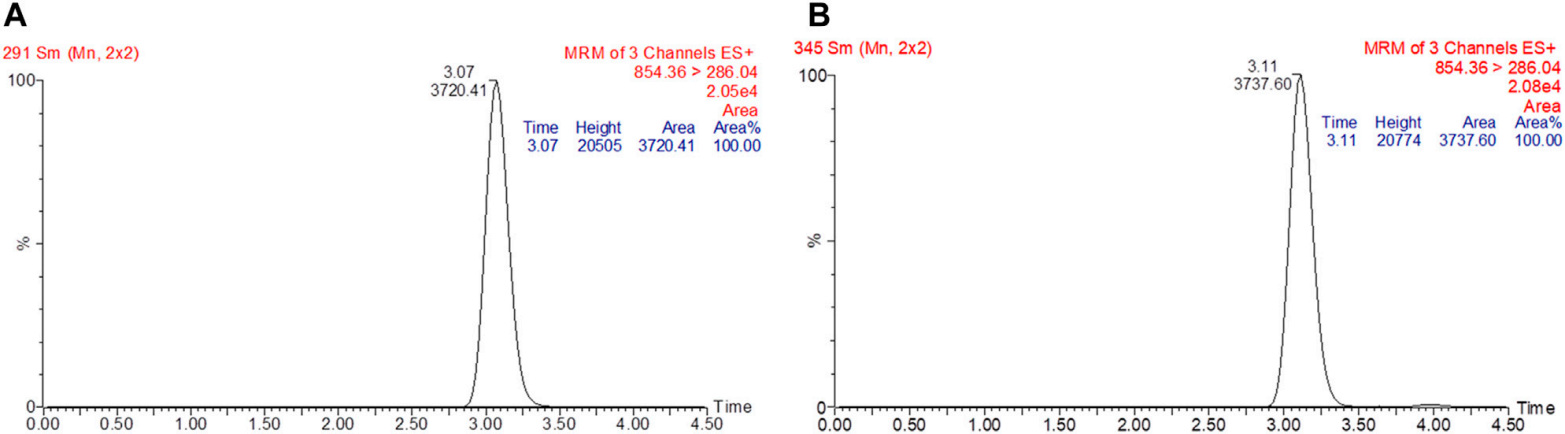
Chromatogram of standard paclitaxel prepared in methanol **(A)**. The retention time for paclitaxel was at 3.07 min with a peak area of 3720.41. Chromatogram of paclitaxel spiked in rat plasma **(B)**. The retention time for paclitaxel was at 3.11 min with a peak area of 3737.60.

The linearity of the calibration curve was established by plotting the peak ratio of paclitaxel. Good linearity was achieved over the concentration range of 5–1,000 ng/ml with a coefficient of estimation (*r* ^2^) of 0.9998. Chromatograms of the standard and paclitaxel-spiked rat plasma are shown in Figures 2A,B. The LOD of paclitaxel was 2 ng/ml with an S/N ratio of 5.3, and the LOQ was 5 ng/ml with an S/N ratio of 33.9. The values of both precision and accuracy were within 15% (Table 1), which is an acceptable criterion. Both the extraction efficiency and matrix effect were within the acceptable limits of >80% (Table 2). These results proved that the LC/MS/MS method was reliable and reproducible; thus, it was used for sample analysis.

**TABLE 1:** Intra-day and inter-day accuracy (%bias) and precision (%RSD) values for quantifying paclitaxel in plasma using an LC/MS/MS method.

Cnormal (ng/ml)	Intra-day (*n* = 6)			Inter-day (*n* = 6)		
	Cobs (ng/ml)	Precision (%)	Accuracy (%)	Cobs (ng/ml)	Precision (%)	Accuracy (%)
5	5.03 ± 0.1	0.9	0.6	4.9 ± 0.1	2.1	−2.0
10	10.67 ± 1.2	10.8	6.7	10.6 ± 0.7	6.1	6.0
50	47.31 ± 4.0	8.5	−5.4	48.1 ± 2.7	5.5	−3.8
100	96.41 ± 5.2	5.4	−3.6	98.9 ± 2.7	2.7	−1.1
500	527.78 ± 24.4	4.6	5.6	493.8 ± 26.5	5.4	−1.2
1,000	1,026.90 ± 121.9	11.9	2.7	998.2 ± 22.9	2.3	−0.2

C normal: Normal concentration.

Cobs: Observed concentration.

Data expressed as the mean ± standard deviation.

Accuracy = (Cobs-Cnormal)/Cnormal × 100; precision = standard deviation/Cobs × 100.

**TABLE 2:** Recovery rate and matrix effect of paclitaxel.

Cnormal (ng/ml)	Spiked in mobile phase (set 1)	Spiked after extraction (set 2)	Spiked before extraction (set 3)	Matrix effect (%)	Recovery rate (%)
10	44 ± 5	38 ± 3	34 ± 2	85	91
100	313 ± 118	275 ± 95	244 ± 0.8	88	89
1,000	2642 ± 600	2341 ± 926	2006 ± 342	89	86

C normal: Normal concentration.

Data are expressed as the mean ± standard deviation (*n* = 3).

Matrix effect = (Set 2/Set 1) × 100, and recovery rate = (Set 3/Set 2) × 100.

### 3.3 Effect of XSLJZT on the Pharmacokinetics of Paclitaxel in Rats

**FIGURE 3:**
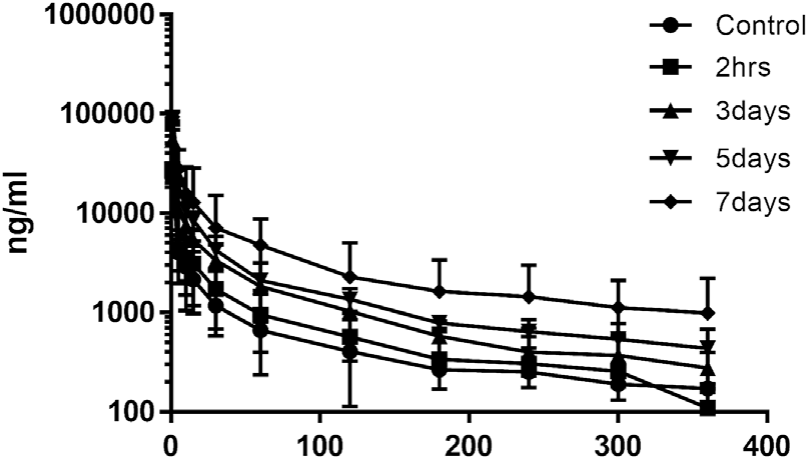
Mean time-concentration curve of paclitaxel in rat blood samples. *n* = 6. The curve shows the change in plasma concentration of paclitaxel in the groups that were pre-treated with XSLJZT for 3, 5, and 7 days.

The effects of XSLJZT on paclitaxel plasma concentrations were evaluated by comparing the changes in PK parameters between the treatment groups and the control group. The time-concentration curve is displayed in Figure 3, and the PK parameters are summarized in Table 3. The results indicated that XSLJZT significantly increased the AUC in the pretreated groups. The highest increase was observed in the 7-days-pretreated group with an AUC of 786.44 ± 193.14 min μg/ml as compared to the control group which had an AUC of 186.19 ± 64.42 min μg/ml. No significant change in the AUC (212.36 ± 138.92 min μg/ml) was observed in the co-treatment group. XSLJZT significantly prolonged the half-life (T_1/2_) from 17.11 ± 2.48 min to 22.77 ± 2.08, 30.75 ± 8.81, 34.74 ± 7.97, and 37.56 ± 4.75 in the co-treatment groups and in the pretreatment groups of 3, 5, and 7 days, respectively. However, the MRT was significantly prolonged only in the pretreatment groups of 5 and 7 days.

**TABLE 3:** Pharmacokinetic properties of paclitaxel (2 mg/kg i.v) with or without XSLJZT (250 mg/kg).

Parameters	Units	Paclitaxel (2 mg/kg)	Pretreatment groups			Co-treatment group
			3 Days	5 Days	7 Days	2 h
AUC	Min µg/mL	186.2 ± 64.4	439.2 ± 89.9^*^	631.7 ± 190.6^*^	786.4 ± 193.1^**^	212.4 ± 138.9
Co	µg/mL	87.9 ± 67.3	111.3 ± 116.1	116.3 ± 74.3	134.7 ± 16.0	65.8 ± 6.5
T_1/2_	Min	17.1 ± 2.5	32.7 ± 7.1^*^	34.7 ± 8.0^*^	37.6 ± 4.8^*^	22.8 ± 2.1^*^
MRT	Min	21.5 ± 9.3	35.5 ± 8.7	38.8 ± 10.4^*^	45.9 ± 11.7^*^	21.4 + 8.3
K10		0.4 ± 0.3	0.3 ± 0.4	0.27 ± 0.04	0.2 ± 0.07	0.4 ± 0.2

Abbreviations: AUC, area under the concentration versus time curve; C0, drug concentration at zero time; T1/2, terminal half-life; K10, elimination constant. (*p < 0.05, **p < 0.001).

### 3.4 Influence of XSLJZT on CYP3A4 Expression

**FIGURE 4:**
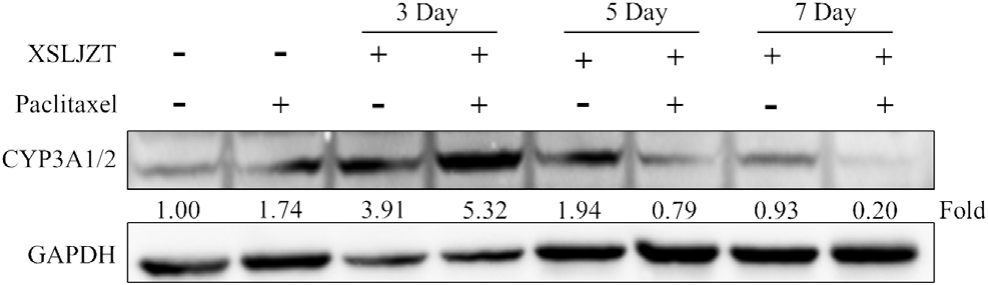
Results showing the inhibitory effect of XSLJZT on CYP3A4 expression in SD rat liver tissues. XSLJZT inhibited the expression of CYP3A4 enzymes with highest effect observed in the 7-days-pretreatment group.

**FIGURE 5:**
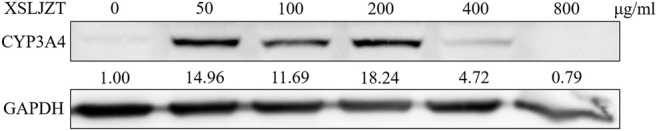
Results showing the inhibitory effect of XSLJZT on CYP3A4 enzyme expression in Hep G2 cells after 24 h incubation period. XSLJZT inhibited CYP3A6 enzyme expression in a dose-dependant manner with the highest effect observed at 800 μg/ml.

**FIGURE 6:**
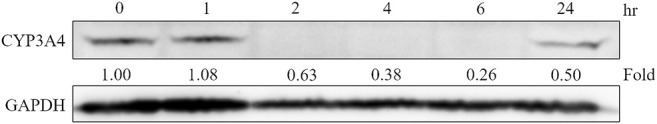
Cells were treated with 800 μg/ml XSLJZT and incubated for different incubation times. The results indicated that XSLJZT inhibited CYP3A4 enzyme expression in a time-dependant manner.

SD rat liver tissues and Hep-G2 cells were used to evaluate the influence of XSLJZT on CYP 3A4 enzymes. XSLJZT inhibited the enzyme expression in both the rat liver tissues and Hep-G2 cells. In the rat liver tissue, XSLJZT inhibited CYP3A1/2 expression starting from 5 days pretreatment. However, the highest effect was observed in the 7 days pretreatment group (Figure 4). In Hep G2 cells, XSLJZT inhibited the expression of CYP3A4 in a dose-and time-dependent manner. The highest inhibition was observed at a concentration of 800 μg/ml XSLJZT (Figures 5, 6).

### 3.5 Inhibitory Effect of XSLJZT on Enzyme Activity in HLMs

**FIGURE 7:**
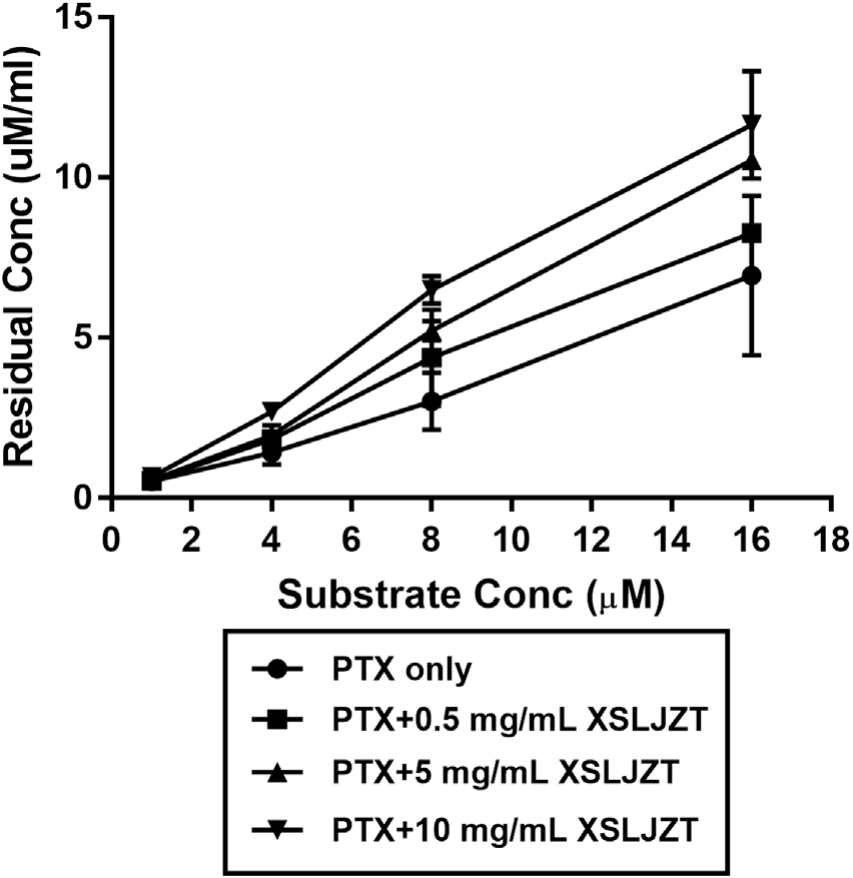
The inhibitory effect of XSLJZT on paclitaxel metabolism in HLMs. Data were expressed at mean ± standard deviation. XLJZT inhibited paclitaxel metabolism in a dose-dependent manner with the highest effect observed in the groups co-incubated with XSLJZT 10 mg/ml.

**FIGURE 8:**
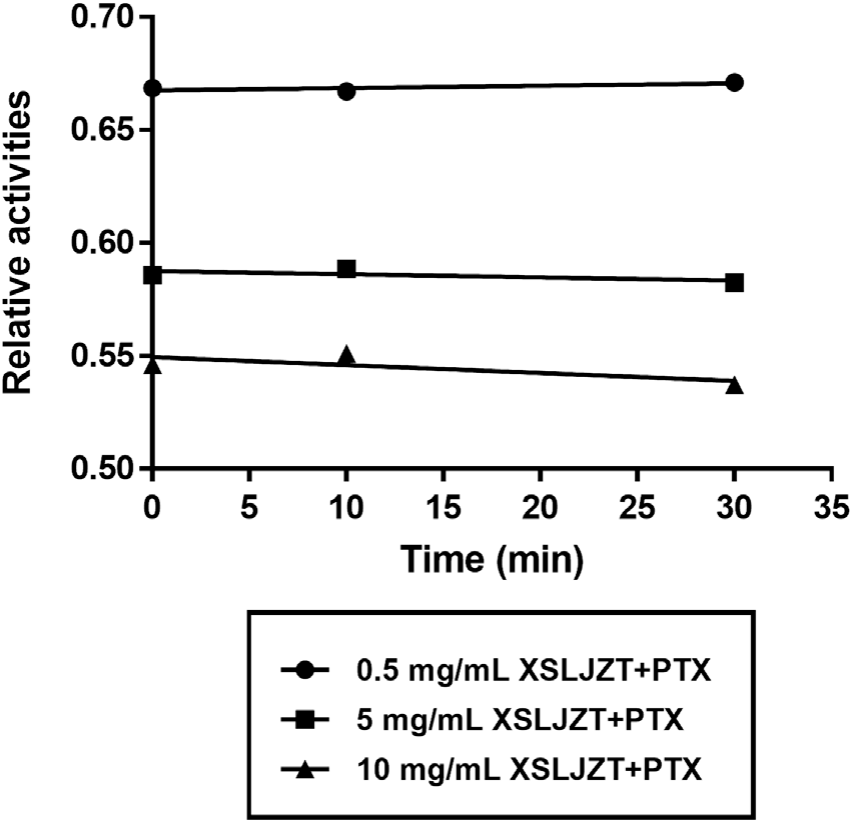
Time-course inhibition of XSLZJT on enzyme activity in HLMs. HLMs were pre-incubated with XSLJZT for 0, 10, and 30 min before the addition of 16 µM paclitaxel. XSLJZT inhibited the enzyme activity in a dose-dependent manner but no time-dependence was observed.

**FIGURE 9:**
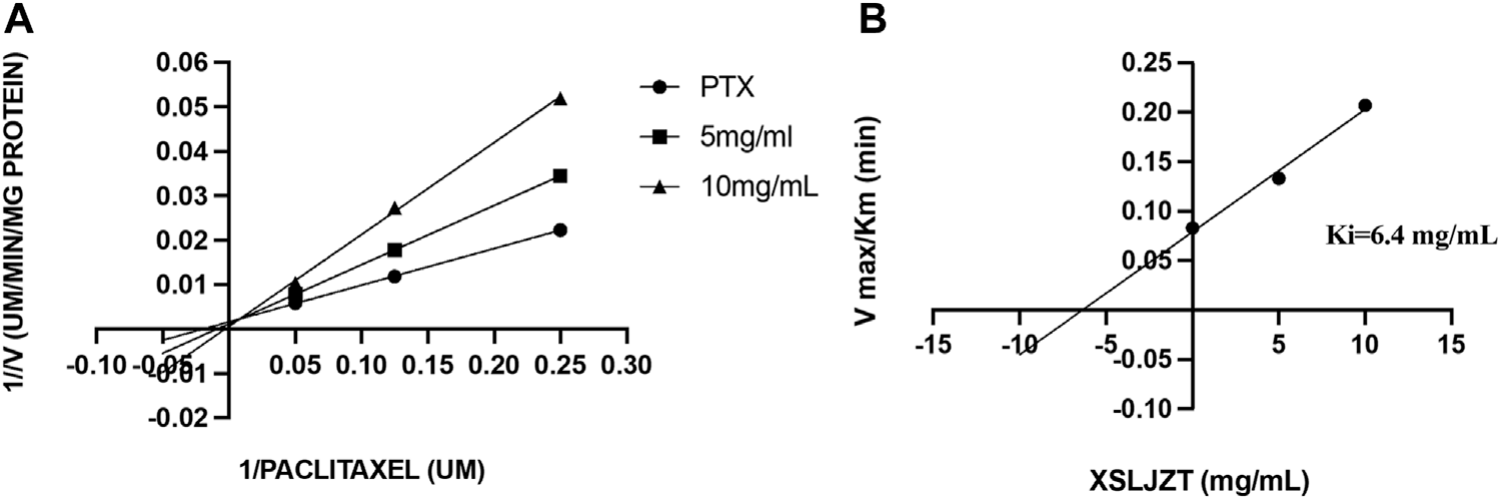
The Lineweaver-Burk plot **(A)** was obtained after different concentrations of paclitaxel (1, 8, and 16 µM) were incubated with different concentrations of XSLJT (5 and 10 mg/ml) for 30 min. XSLJZT inhibited the enzyme activity by a competitive inhibition method. The second plot of slope from Lineweaver-Burk plots **(B)** was obtained and XSLJZT competitively inhibited XSLJZT with a Ki value of 15 µM.

HLMs were incubated with paclitaxel (16, 8, 4, and 1 µM) with or without XSLJZT (0.5, 5, and 10 mg/ml) for 30 min. The residual paclitaxel concentration significantly increased in the groups where HLMs were incubated with both paclitaxel and XSLJZT as compared to the control group, which was incubated with paclitaxel only. This indicated that XSLJZT inhibited paclitaxel metabolism, which in turn increased its concentration (Figure 7). To determine the influence of XSLJT on enzyme activity at different incubation times, the HLMs were pre-incubated with different concentrations of XSLJZT (0.5, 5, and 10 mg/ml) for 0, 10, and 30 min. No significant difference was observed with the different pre-incubation times, indicating that XSLJZT did not significantly inhibit XSLJZT in a time-dependent manner (Figure 8). To understand the inhibition kinetics, different concentrations of XSLJZT (5 and 10 mg/ml) were incubated with different concentrations of paclitaxel (4, 8, and 16 µM). A Lineweaver-Burk plot and a secondary plot indicated that XSLJZT inhibited the enzyme activity in HLMs by competitive inhibition with a Ki value of 6.4 mg/ml (Figures 9A,B).

## 4 Discussion

Different species metabolize paclitaxel differently. 6α-OHP is the major paclitaxel metabolite formulated in humans which is seconded by C3′α-OHP. 6α-OHP is formulated by CYP2C8 enzymes in humans which is not available in rats. In both rats and humans, paclitaxel is metabolized to C3′α-OHP by CYP3A enzymes. In humans C3′α-OHP is formulated by isoform CYP3A4 and in rats it is formulated by isoform CYP3A2/1. The rats CYP3A1/2 enzymes are orthologous to the human CYP3A4 enzymes (Václavíková et al., 2003; Vaclavikova et al., 2004; Sun et al., 2016). The influence of XSLJZT on CYP3A enzymes was evaluated in both Hep-G2 cells and rat liver tissues. In the rat liver tissue, the animals were pre-treated with XSLJZT prior to the experiment. XSLJZT inhibited CYP3A1/2 enzymes expression differently with variations in pre-treatment periods. In the Hep-G2 cells, XSLJZT inhibited CYP3A4 enzymes expression in a dose and time dependent manner.

In the rat blood plasma, XSLJZT increased paclitaxel AUC differently with variation in co-treatment and pre-treatment periods. In line with the animal study, western blot results indicated a variation in the reduction of CYP3A1/2 enzyme expression with differences in pre-treatment days. Similar results were observed in the HLM, where XSLJZT inhibited enzymes activities which in turn decrease paclitaxel metabolism and increased its residual concentration. In line with the enzyme activity study, XSLJZT inhibited the expression on CYP3A4 enzymes in Hep-G2 cells.

XSLJZT is a herbal formula that contains a high polyphenol content. Liu *et al.* reported that glycyrrhizin and liquiritin from *Glycyrrhiza uralensis* (*Gan Cao*), quercetrin, and hesperidin from *Citrus reticulata* (*Chen Pi*), ginsenoside Rg1, ginsenoside Re, ginsenoside Rb1 from *Panax ginseng* (*Ren Shen*), 6-gingerol and 6-shogaol from *Zingiber officinale*, and costunolide and dehydrocostus lactone from *Aucklandia lappa* (*Mu Xiang*) could be detected in XSLJZT using HPLC-MS (Liu et al., 2017). Polyphenols can potentially influence the cytotoxicity of chemotherapeutic drugs either by additive or synergistic effects, which can result in toxicity (Mahbub et al., 2015; Klimaszewska-Wisniewska et al., 2016). They can also affect the metabolizing enzymes either by direct polyphenol enzyme binding or by affecting the protein expression of the enzymes. In previous reports, it was observed that most polyphenols inhibit the expression of the major metabolizing enzymes in the body (CYP-450 enzymes), which can affect the metabolism of co-administered substrate drugs (Korobkova, 2015; Yang et al., 2019).

Studies have reported an increase in the use of herbal medicines among cancer patients in order to minimize the side effects of chemotherapy and improve the general health (Yin et al., 2013). Although herbal medicines can minimize the side effects of chemotherapy, it is important to assess the potential herb-drug PK interactions. In a clinical setting, cancer patients can take herbal medicines before, during, and or after chemotherapy. The combination of herbal medicines and chemotherapy can potentially result in herb-drug interactions. Therefore, in order to understand the potential influence of XSLJZT on paclitaxel pharmacokinetics in clinics, SD rats were classified into control, pretreatment, and co-treatment groups (Koçaşlı and Demircan, 2017; Li et al., 2020). XSLJZT differently influenced paclitaxel PK parameters, with variation in pretreatment days. It increased paclitaxel AUC for 2-, 3-, and 4-fold after 3, 5, and 7 days of pretreatment, respectively. However, it did not significantly influence the AUC of paclitaxel in the co-treatment group. XSLJZT significantly prolonged the half-life of paclitaxel in both pre-and co-treatment groups, with the highest increase in the 7-days pretreatment group.

To investigate the influence of XSLJZT on paclitaxel metabolism in humans, an enzyme kinetic study was conducted using HLMs. Human liver microsomes are the dominant systems used *in vitro* to understand drug metabolism and can be used to easily understand the effect of herbal medicines on chemotherapeutic metabolism in the clinic (Zhang et al., 2015). XSLJZT increased the residual paclitaxel concentration in a dose-dependent manner, with the highest increase observed in the group co-incubated with 10 mg/ml XSLJZT. It competitively inhibited enzyme activity in a dose-dependent manner. However, no significant time-dependency was observed.

XSLJZT is a herb commonly used by breast cancer patients. It contains a high content of polyphenols, which could possibly result in herb-drug PK interactions (Kaspera and Croteau, 2006). *Panax ginseng* (Ren Shen) in XSLJZT contains ginsenosides that specifically inhibit metabolism and decrease CYP3A4 enzyme activity, gene, and protein expression, and *Glycyrrhiza uralensis* (Gan Cao) can inhibit CYP2C8 (Yu et al., 2011; Li et al., 2017). In this study, XSLJZT increased paclitaxel AUC in rat blood samples, inhibited CYP 3A enzymes expression and inhibited paclitaxel metabolism in human liver microsomes but the effects of individual XSLJZT herbs on paclitaxel were not evaluated.

## 5 Conclusion

In this study, the influence of XSLJZT on paclitaxel was assessed. XSLJZT increased paclitaxel AUC by 2-, 3-, and 4-fold in the groups pretreated for 3, 5, and 7 days, respectively. However, no significant influence was observed in the co-treated groups. In line with the animal study, XSLJZT inhibited paclitaxel metabolism in HLM which resulted in the increase in paclitaxel residual concentration. Western blot results indicated a reduction in CYP3A1/2 enzyme expression in rat liver tissues and CYP3A4 enzymes in Hep-G2 cells. The inhibitory effects of XSLJZT on CYP3A enzymes can potentially result in paclitaxel herb-drug interactions. In the pharmacokinetic results, it was observed that XSLJZT influenced paclitaxel parameters differently with variations in pre-treatment days and in the rat liver tissue, XSLJZT inhibited CYP3A1/2 enzymes expression differently with variations in pre-treatment days. This indicates that XSLJZT can clinically affect paclitaxel differently with differences in pretreatment and co-treatment regimens.

Although XSLJZT significantly increased paclitaxel AUC and inhibited CYP3A1/2 enzymes in the pretreatment groups, no significant difference was detected in the co-treatment group. This could indicate that XSLJZT can be safely co-administered with paclitaxel on the same treatment day but should be monitored in longer pre-treatment durations. This study serves as a foundation for guidance when XSLJZT is co-administered with paclitaxel and other chemotherapeutics metabolized via a similar pathway. Different species metabolize paclitaxel differently therefore to fully establish the effects of XSLJZT on paclitaxel in clinic, further studies should be carried out with human experiments.

## Data Availability

The original contributions presented in the study are included in the article/Supplementary Material, further inquiries can be directed to the corresponding authors.

## References

[B1] Bernabeu E. Cagel M. Lagomarsino E. Moretton M. Chiappetta D. A. (2017). Paclitaxel: what Has Been Done and the Challenges Remain Ahead. Int. J. Pharm. 526 (1-2), 474–495. 10.1016/j.ijpharm.2017.05.016 28501439

[B2] Boussios S. Pentheroudakis G. Katsanos K. Pavlidis N. (2012). Systemic Treatment-Induced Gastrointestinal Toxicity: Incidence, Clinical Presentation and Management. Ann. Gastroenterol. 25 (2), 106–118. 24713845 PMC3959393

[B3] Cheng X. Lv X. Qu H. Li D. Hu M. Guo W. (2017). Comparison of the Inhibition Potentials of Icotinib and Erlotinib against Human UDP-Glucuronosyltransferase 1A1. Acta Pharm. Sin. B. 7 (6), 657–664. 10.1016/j.apsb.2017.07.004 29159025 PMC5687316

[B4] Cheng Y. Y. Hsieh C. H. Tsai T. H. (2018). Concurrent Administration of Anticancer Chemotherapy Drug and Herbal Medicine on the Perspective of Pharmacokinetics. J. Food Drug Anal. 26 (2S), S88–S95. 10.1016/j.jfda.2018.01.003 29703390 PMC9326883

[B5] Cui H. M. Zhang Q. Y. Wang J. L. Chen J. L. Zhang Y. L. Tong X. L. (2014). *In Vitro* studies of Berberine Metabolism and its Effect of Enzyme Induction on HepG2 Cells. J. Ethnopharmacol. 158 Pt A, 388–396. 10.1016/j.jep.2014.10.018 25456436

[B6] Fasinu P. S. Rapp G. K. (2019). Herbal Interaction with Chemotherapeutic Drugs-A Focus on Clinically Significant Findings. Front. Oncol. 9, 1356. 10.3389/fonc.2019.01356 31850232 PMC6901834

[B7] Gallego-Jara J. Lozano-Terol G. Sola-Martínez R. A. Cánovas-Díaz M. de Diego Puente T. (2020). A Compressive Review about Taxol: History and Future Challenges. Molecules 25 (24), 5986. 10.3390/molecules25245986 PMC7767101 33348838

[B8] Health, U.D.o., H. Services (2001). Bioanalytical Method Validation, Guidance for Industry. Available at: http://www.fda.gov./cder/guidance/4252fnl.Htm.

[B9] Hendrikx J. J. Lagas J. S. Rosing H. Schellens J. H. Beijnen J. H. Schinkel A. H. (2013). P-glycoprotein and Cytochrome P450 3A Act Together in Restricting the Oral Bioavailability of Paclitaxel. Int. J. Cancer 132 (10), 2439–2447. 10.1002/ijc.27912 23090875

[B10] Huang K. C. Yen H. R. Chiang J. H. Su Y. C. Sun M. F. Chang H. H. (20172017). Chinese Herbal Medicine as an Adjunctive Therapy Ameliorated the Incidence of Chronic Hepatitis in Patients with Breast Cancer: A Nationwide Population-Based Cohort Study. Evid. Based Complement. Alternat. Med. 2017, 1052976. 10.1155/2017/1052976 PMC5682887 29234362

[B11] Kaspera R. Croteau R. (2006). Cytochrome P450 Oxygenases of Taxol Biosynthesis. Phytochem. Rev. 5 (2-3), 433–444. 10.1007/s11101-006-9006-4 20622990 PMC2901147

[B12] Klimaszewska-Wisniewska A. Halas-Wisniewska M. Tadrowski T. Gagat M. Grzanka D. Grzanka A. (2016). Paclitaxel and the Dietary Flavonoid Fisetin: a Synergistic Combination that Induces Mitotic Catastrophe and Autophagic Cell Death in A549 Non-small Cell Lung Cancer Cells. Cancer Cel. Int. 16, 10. 10.1186/s12935-016-0288-3 PMC4754822 26884726

[B13] Kocasli S. Demircan Z. (2017). Herbal Product Use by the Cancer Patients in Both the Pre and post Surgery Periods and during Chemotherapy. Ajtcam 14 (2), 325–333. 10.21010/ajtcam.v14i2.34 28573249 PMC5446458

[B14] Korobkova E. A. (2015). Effect of Natural Polyphenols on CYP Metabolism: Implications for Diseases. Chem. Res. Toxicol. 28 (7), 1359–1390. 10.1021/acs.chemrestox.5b00121 26042469

[B15] Lai J. N. Wu C. T. Wang J. D. (2012). Prescription Pattern of Chinese Herbal Products for Breast Cancer in Taiwan: a Population-Based Study. Evid. Based Complement. Alternat. Med. 2012, 891893. 10.1155/2012/891893 22685488 PMC3368194

[B16] Lee R. T. Kwon N. Wu J. To C. To S. Szmulewitz R. (2021). Prevalence of Potential Interactions of Medications, Including Herbs and Supplements, before, during, and after Chemotherapy in Patients with Breast and Prostate Cancer. Cancer 127 (11), 1827–1835. 10.1002/cncr.33324 33524183

[B17] Lee Y. W. Chen T. L. Shih Y. R. Tsai C. L. Chang C. C. Liang H. H. (2014). Adjunctive Traditional Chinese Medicine Therapy Improves Survival in Patients with Advanced Breast Cancer: a Population-Based Study. Cancer 120 (9), 1338–1344. 10.1002/cncr.28579 24496917

[B18] Li G. Simmler C. Chen L. Nikolic D. Chen S. N. Pauli G. F. (2017). Cytochrome P450 Inhibition by Three Licorice Species and Fourteen Licorice Constituents. Eur. J. Pharm. Sci. 109, 182–190. 10.1016/j.ejps.2017.07.034 28774812 PMC5656517

[B19] Li H. Tang Y. Wei W. Yin C. Tang F. (2021). Effects of Xiaochaihu Decoction on the Expression of Cytochrome P450s in Rats. Exp. Ther. Med. 21 (6), 588. 10.3892/etm.2021.10020 33850560 PMC8027731

[B20] Li S. So T. H. Tang G. Tan H. Y. Wang N. Ng B. F. L. (2020). Chinese Herbal Medicine for Reducing Chemotherapy-Associated Side-Effects in Breast Cancer Patients: a Systematic Review and Meta-Analysis. Front. Oncol. 10, 599073. 10.3389/fonc.2020.599073 33363030 PMC7756083

[B21] Liu J. H. Cheng Y. Y. Hsieh C. H. Tsai T. H. (2017). Identification of a Multicomponent Traditional Herbal Medicine by HPLC-MS and Electron and Light Microscopy. Molecules 22 (12), 2242. 10.3390/molecules22122242 PMC6150010 29244753

[B22] Liu J. H. Cheng Y. Y. Hsieh C. H. Tsai T. H. (2018). The Herb-Drug Pharmacokinetic Interaction of 5-Fluorouracil and its Metabolite 5-Fluoro-5,6-Dihydrouracil with a Traditional Chinese Medicine in Rats. Int. J. Mol. Sci. 19 (1), 25. 10.3390/ijms19010025 PMC5795976 29295501

[B23] Lo L. C. Chen C. Y. Chen S. T. Chen H. C. Lee T. C. Chang C. S. (2012). Therapeutic Efficacy of Traditional Chinese Medicine, Shen-Mai San, in Cancer Patients Undergoing Chemotherapy or Radiotherapy: Study Protocol for a Randomized, Double-Blind, Placebo-Controlled Trial. Trials 13, 232. 10.1186/1745-6215-13-232 23206440 PMC3543266

[B24] Mahbub A. A. Le Maitre C. L. Haywood-Small S. L. Cross N. A. Jordan-Mahy N. (2015). Polyphenols Act Synergistically with Doxorubicin and Etoposide in Leukaemia Cell Lines. Cell Death Discov. 1, 15043. 10.1038/cddiscovery.2015.43 27551472 PMC4979421

[B25] McDonnell A. M. Dang C. H. (2013). Basic Review of the Cytochrome P450 System. J. Adv. Pract. Oncol. 4 (4), 263–268. 10.6004/jadpro.2013.4.4.7 25032007 PMC4093435

[B26] Ohnishi S. Takeda H. (2015). Herbal Medicines for the Treatment of Cancer Chemotherapy-Induced Side Effects. Front. Pharmacol. 6, 14. 10.3389/fphar.2015.00014 25713534 PMC4322614

[B27] Shih Y. S. Tsai C. H. Li T. C. Lai H. C. Wang K. T. Liao W. L. (2019). The Effect of Xiang-Sha-Liu-Jun-Zi Tang (XSLJZT) on Irritable Bowel Syndrome: a Randomized, Double-Blind, Placebo-Controlled Trial. J. Ethnopharmacol. 238, 111889. 10.1016/j.jep.2019.111889 31009707

[B29] Smith P. J. Clavarino A. Long J. Steadman K. J. (2014). Why Do Some Cancer Patients Receiving Chemotherapy Choose to Take Complementary and Alternative Medicines and what Are the Risks? Asia Pac. J. Clin. Oncol. 10 (1), 1–10. 10.1111/ajco.12115 23910177

[B30] Sun Z. Zhang Z. Ji M. Yang H. Cromie M. Gu J. (2016). BDE47 Induces Rat CYP3A1 by Targeting the Transcriptional Regulation of miR-23b. Sci. Rep. 6, 31958. 10.1038/srep31958 27546062 PMC4992956

[B31] Tekade R. K. D'Emanuele A. Elhissi A. Agrawal A. Jain A. Arafat B. T. (2013). Extraction and RP-HPLC Determination of Taxol in Rat Plasma, Cell Culture and Quality Control Samples. J. Biomed. Res. 27 (5), 394–405. 10.7555/JBR.27.20120123 24086173 PMC3783825

[B32] Vaclavikova R. Soucek P. Svobodova L. Anzenbacher P. Simek P. Guengerich F. P. (2004). Different *In Vitro* Metabolism of Paclitaxel and Docetaxel in Humans, Rats, Pigs, and Minipigs. Drug Metab. Dispos. 32 (6), 666–674. 10.1124/dmd.32.6.666 15155559

[B33] Václavíková R. Horský S. Šimek P. Gut I. (2003). Paclitaxel Metabolism in Rat and Human Liver Microsomes Is Inhibited by Phenolic Antioxidants. Naunyn-schmiedeberg's Arch. Pharmacol. 368 (3), 200–209. 10.1007/s00210-003-0781-9 12920504

[B34] Wattanachai N. Polasek T. M. Heath T. M. Uchaipichat V. Tassaneeyakul W. Tassaneeyakul W. (2011). In Vitro-In Vivo Extrapolation of CYP2C8-Catalyzed Paclitaxel 6α-Hydroxylation: Effects of Albumin on *In Vitro* Kinetic Parameters and Assessment of Interindividual Variability in Predicted Clearance. Eur. J. Clin. Pharmacol. 67 (8), 815–824. 10.1007/s00228-011-1001-z 21305272

[B35] Xiao H. Liu L. Ke S. Zhang Y. Zhang W. Xiong S. (2021). Efficacy of Xiang-Sha-Liu-Jun-Zi on Chemotherapy-Induced Nausea and Vomiting. Medicine (Baltimore) 100 (19), e25848. 10.1097/MD.0000000000025848 34106627 PMC8133094

[B36] Xiao Y. Liu Y. Y. Yu K. Q. Ouyang M. Z. Luo R. Zhao X. S. (2012). Chinese Herbal Medicine Liu Jun Zi Tang and Xiang Sha Liu Jun Zi Tang for Functional Dyspepsia: Meta-Analysis of Randomized Controlled Trials. Evid. Based Complement. Alternat. Med. 2012, 936459. 10.1155/2012/936459 23304226 PMC3530827

[B37] Yang L. Wang Y. Xu H. Huang G. Zhang Z. Ma Z. (20192019). *Panax Ginseng* Inhibits Metabolism of Diester Alkaloids by Downregulating CYP3A4 Enzyme Activity via the Pregnane X Receptor. Evid. Based Complement. Alternat. Med. 2019, 3508658. 10.1155/2019/3508658 PMC6463675 31057647

[B38] Yin S. Y. Wei W. C. Jian F. Y. Yang N. S. (2013). Therapeutic Applications of Herbal Medicines for Cancer Patients. Evid. Based Complement. Alternat. Med. 2013, 302426. 10.1155/2013/302426 23956768 PMC3727181

[B39] Yu C. Chai X. Yu L. Chen S. Zeng S. (2011). Identification of Novel Pregnane X Receptor Activators from Traditional Chinese Medicines. J. Ethnopharmacol. 136 (1), 137–143. 10.1016/j.jep.2011.04.022 21524698

[B40] Zanger U. M. Schwab M. (2013). Cytochrome P450 Enzymes in Drug Metabolism: Regulation of Gene Expression, Enzyme Activities, and Impact of Genetic Variation. Pharmacol. Ther. 138 (1), 103–141. 10.1016/j.pharmthera.2012.12.007 23333322

[B41] Zhang H. Gao N. Tian X. Liu T. Fang Y. Zhou J. (2015). Content and Activity of Human Liver Microsomal Protein and Prediction of Individual Hepatic Clearance *In Vivo* . Sci. Rep. 5, 17671. 10.1038/srep17671 26635233 PMC4669488

